# Biological Sequence Representation Methods and Recent Advances: A Review

**DOI:** 10.3390/biology14091137

**Published:** 2025-08-27

**Authors:** Hongwei Zhang, Yan Shi, Yapeng Wang, Xu Yang, Kefeng Li, Sio-Kei Im, Yu Han

**Affiliations:** 1 Faculty of Applied Sciences, Macao Polytechnic University, Macau 999078, China; hongwei.zhang@mpu.edu.mo (H.Z.); xuyang@mpu.edu.mo (X.Y.); marcusim@mpu.edu.mo (S.-K.I.); 2 State Key Laboratory of Networking and Switching Technology, Beijing University of Posts and Telecommunications, Beijing 100876, China; 3 Center for Artificial Intelligence Driven Drug Discovery, Faculty of Applied Sciences, Macao Polytechnic University, Macau 999078, China; kefengl@mpu.edu.mo; 4 Faculty of Civil Engineering, Southwest Forestry University, Kunming 650224, China; hanyu@swfu.edu.cn

**Keywords:** biological sequence, computational, word embedding, large language model, machine learning

## Abstract

Biological-sequence representation methods are pivotal for advancing machine learning in computational biology, transforming nucleotide and protein sequences into formats that enhance predictive modeling and downstream task performance. This review categorizes these methods into three developmental stages: computational-based, word embedding-based, and large language model (LLM)-based, detailing their principles, applications, and limitations. Computational-based methods, such as k-mer counting and position-specific scoring matrices (PSSM), extract statistical and evolutionary patterns to support tasks like motif discovery and protein–protein interaction prediction. Word embedding-based approaches, including Word2Vec and GloVe, capture contextual relationships, enabling robust sequence classification and regulatory element identification. Advanced LLM-based methods, leveraging Transformer architectures like ESM3 and RNAErnie, model long-range dependencies for RNA structure prediction and cross-modal analysis, achieving superior accuracy. However, challenges persist, including computational complexity, sensitivity to data quality, and limited interpretability of high-dimensional embeddings. Future directions prioritize integrating multimodal data (e.g., sequences, structures, and functional annotations), employing sparse attention mechanisms to enhance efficiency, and leveraging explainable AI to bridge embeddings with biological insights. These advancements promise transformative applications in drug discovery, disease prediction, and genomics, empowering computational biology with robust, interpretable tools.

## Simple Summary

This paper investigates how to convert biological sequences (protein and nucleotide sequences) into a format that computers can understand to better advance computational biology research. Our goal is to explain the principles and technical details of these methods, mainly to illustrate their application areas and advantages and disadvantages. We reviewed early techniques that relied on counting patterns, later methods that imitated language-processing techniques to capture context, and state-of-the-art methods based on large models in recent years. Our results show that new methods are more accurate but require advanced computing power. The development and improvement of these methods will help scientists design effective drugs, predict diseases, and reveal the connection between genetic material and proteins. We conduct a fundamental research work, this review can provide useful guidance and help for researchers in computational biology, especially those new to the field.

## 1. Introduction

The primary aim of biological-sequence representation methods is to convert nucleotide and protein sequences into formats that can be interpreted by computing systems. Form the backbone of computational biology, it provides the possibility for efficient processing and in-depth analysis of complex biological data. The emergence of large-scale genome sequencing, especially since the implementation of the Human Genome Project [1,2,3,4], has generated vast and complex datasets. Utilizing these datasets to uncover new biological insights is a significant and influential effort [5,6,7,8,9,10,11]. The biological-sequence representation method effectively captures sequence information from multiple dimensions, including statistical patterns, physical and chemical properties, structural features, contextual relationships between nucleotides and amino acids, and long-range dependencies. By providing a comprehensive and robust framework for data representation, these methods have laid a solid foundation for downstream machine learning applications, thereby advancing biomedical research.

The evolution of sequence representation methods can be categorized into three developmental stages: Early computational-based methods, using methods like k-mer analysis and position-specific scoring matrices (PSSM) extracted statistical (e.g., nucleotide composition,), physicochemical properties (e.g., hydrophobicity, polarity, and charge of proteins) and evolutionary features, often paired with shallow machine learning models like support vector machine (SVM) [12] and random forest (RF) [13] for genome assembly, structure prediction and protein–protein interaction (PPI) prediction [14,15,16]. Word embedding-based methods such as Word2Vec and ProtVec, leveraging deep learning methods, convolutional neural network (CNN) [17] and long short-term memory (LSTM) [18], capture contextual relationship for sequence classification, protein function annotation [19,20]. Recent advances leverage large language model (LLM) based methods, employing attention mechanisms [21] and models like AlphaFold3 and RoseTTAFold All-Atom, model complex sequence–struture–function relationships for structure prediction and functional annotation [22,23], these methods achieve high accuracy but come with increased computational demands. These methods address diverse biological tasks by meeting specific representation needs: computational-based methods excel in efficient local pattern capture for tasks like sequence classification, word embedding-based methods model contextual relationships for functional annotation, and LLM-based methods capture long-range dependencies for complex tasks like 3D structure prediction.

**Figure 1:**
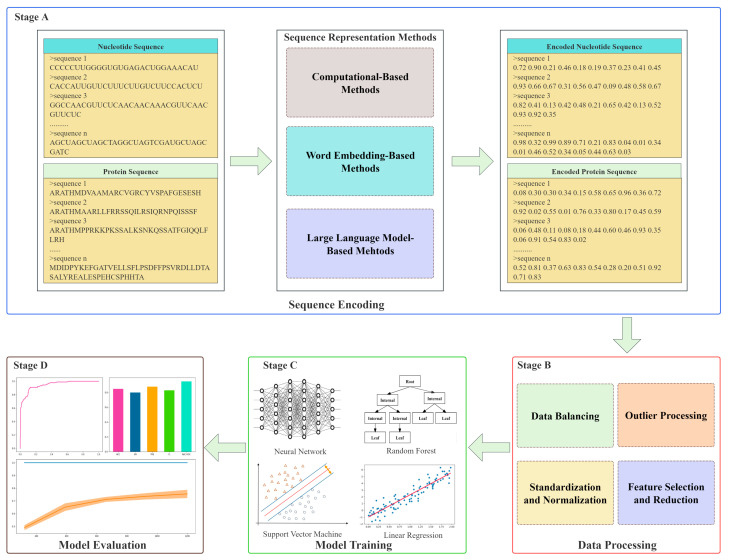
Shows a process framework for biological data analysis based on machine learning. The direction indicated by the arrow illustrates the sequential process of the framework. The process is divided into four stages: Stage A involves encoding input sequence data, transforming row biological sequence into a suitable format for analysis. Stage B encompasses preprocessing steps, including outlier handling, normalization, feature selection, dimensionality reduction, and data balancing; to enhance data quality and consistency. In stage C, ML methods such as neural network (NN), RF, SVM, and linear regression (LR) are employed for model training, enabing the development of predictive models. Stage D evaluates model performance using metrics, including accuracy, precision, recall, F1-score, and area under the receiver operating characteristic curve (AUC-ROC), to assess predictive reliability and generalizability for computational biology tasks like sequence classification and function prediction.

Despite these advancements, challenges persist, including computational complexity, data quality, and model interpretability. Figure 1 illustrates a machine learning framework for biological data analysis, outlining the stages of data encoding, preprocessing, model training, and evaluation.

This review systematically traces the development of these methods, highlighting their principles, applications, and limitations. Subsequent sections detail computational-based methods (Section 2), word embedding-based methods (Section 3), LLM-based techniques (Section 4), challenges and future directions (Section 5), and conclude with insights into their transformative potential (Section 6).

## 2. Computational-Based Methods

Computational-based methods represent the earliest stage of biological-sequence representation, focusing on statistical, physicochemical properties, and structural feature extraction from nucleotide and protein sequences. This paper reviews thirteen commonly used methods, categorized into five groups as shown in Table 1.

### 2.1. K-Mer-Based Methods

The k-mer-based methods, a cornerstone of computational-based methods, transform biological sequences into numerical vectors by counting k-mer frequencies. These methods capture local sequence patterns through statistical analysis of contiguous and gapped k-mers [24,25].

#### 2.1.1. Overview

k-mer methods encode biological sequences by counting the frequencies of k-mers, producing vectors with dimensions determined by the sequence alphabet size ($\left|\Sigma \right|=4$ for nucleotides, $\left|\Sigma \right|=20$ for proteins) and k value. For example, nucleotide sequences yield 4-dimensional vectors for mononucleotide composition (MNC) (k=1), 16-dimensional for dinucleotides composition (DNC) (k=2), and 64-dimensional for trinucleotides composition (TNC) (k=3), while protein sequences produce 20, 400, and 8000 dimensions for amino acid composition (AAC), dipeptides composition (DPC), and tripeptides composition (TPC), respectively. Extending this approach, gapped k-mer [26] methods introduce gaps within subsequences, enabling the capture of non-contiguous patterns critical for regulatory sequence analysis. The gkm kernel enhances this by measuring sequence similarity through gapped k-mer frequencies, using efficient tree-based data structures to manage high-dimensional feature spaces. These methods provide robust features for machine learning, with encoding length balancing predictive power and computational cost. Detailed mathematical formulations are provided in Supplementary Materials (Equations (S1)–(S4)).

#### 2.1.2. Applications

Due to their simplicity, flexibility, and ability to capture biologically significant sequence patterns [27,28], the k-mer-based methods excel in sequence comparison, genome assembly, sequence classification, and motif discovery by encoding the frequency of short subsequences. For nucleotide sequences, facilitating the identification of functional sequence elements in regulatory DNA, and population genetic analyses [29,30]. Gapped k-mer methods enhance these capabilities by modeling non-adjacent sequence patterns, enabling regulatory sequence prediction, such as transcription factor binding sites and variant effect prediction, especially in predicting the impact of non-coding variants on gene expression and disease risk, these applications provide a new perspective for understanding how variations affect gene expression regulation and disease-related variations. [31,32]. For protein sequences, gapped k-mers aid subcellular localization prediction (e.g., cytoplasm, nucleus) and functional prediction of proteins like antioxidant proteins by capturing discontinuous physicochemical patterns [33,34]. Gapped k-mers support protein function prediction by identifying hemolytic or antimicrobial peptides, and PPI analysis by capturing compositional and local sequence order information [35,36]. These methods integrate seamlessly with machine learning models, such as SVM, RF, and deep neural networks, to achieve robust predictive performance.

#### 2.1.3. Advantages and Limitations

Their key advantages include the flexibility to adjust the k value, balancing fine-grained local patterns (small k) with broader sequence contexts (larger k), and their straightforward implementation, which supports diverse computational biology applications. However, challenges include high-dimensional feature spaces, particularly for larger k values or gapped k-mers, which can lead to sparsity in large-scale datasets, and parameter sensitivity (e.g., k value or gap size), requiring careful optimization [28]. Feature selection or dimensionality reduction techniques, such as principal component analysis, are critical to improving computational efficiency and scalability in computational biology tasks.

### 2.2. Group-Based Methods

Group-based methods first group sequence elements (nucleotides or amino acids) based on physicochemical properties (such as hydrophobicity, polarity, charge), analyze the position, combination, and frequency of the grouped patterns, and generate low-dimensional and biologically significant feature vectors to represent sequences. Compared with k-mer methods, group-based methods have significant advantages in dimension control, biological relevance, and computational efficiency.

#### 2.2.1. Overview

The Composition, Transition, and Distribution (CTD) [37] groups amino acids into three categories—polar, neutral, and hydrophobic [38] (Supplementary Materials Table S1)—producing a fixed 21-dimensional vector. This includes 3 composition features (group frequencies), 3 transition features (frequencies of switches between groups, e.g., polar to hydrophobic), and 15 distribution features (positions of groups at 25%, 50%, 75%, and 100% of the sequence). Another method, the Conjoint Triad (CT) [36] groups amino acids into seven categories based on properties like dipole and side chain volume, forming triads of three consecutive amino acids. This results in a 343-dimensional vector capturing the frequency of each triad type (Supplementary Materials Figure S1). CTD produces a 21-dimensional vector, while CT produces a 343-dimensional vector. This reflects a trade-off between capturing detailed sequence patterns and maintaining computational tractability, making these methods suitable for tasks requiring biologically relevant features. Detailed mathematical formulations are provided in Supplementary Materials (Equations (S5)–(S7)).

#### 2.2.2. Applications

Group-based methods, including CTD and CT, excel in protein function prediction, sequence analysis, and PPI prediction by leveraging physicochemical properties to generate biologically relevant features. For instance, DeepTP utilized CTD features to predict thermophilic proteins in Thermus thermophilus, achieving 87.2% accuracy in cross-validation datasets, supporting the identification of thermally stable enzymes for industrial applications [39]. Similarly, PreTP-Stack employed CTD to classify therapeutic peptides, including anticancer peptides, achieving an Area Under the Curve (AUC) of 99.0% in multiple datasets, contributing to novel cancer therapy development [40]. CT enhances these capabilities by capturing triad-based patterns, improving accuracy in protein function annotation and PPI prediction across diverse datasets [36,41].

#### 2.2.3. Advantages and Limitations

Group-based methods face challenges, including the need for parameter optimization (e.g., grouping criteria for amino acids), which increases computational complexity, and sparsity in longer sequences, particularly for CTD and CT fixed-dimensional vectors, necessitating feature selection to support tasks like protein function prediction. The limited exploration of CTD in subcellular localization suggests untapped potential for enhancing predictive accuracy in such tasks.

### 2.3. Correlation-Based Methods

Correlation-based methods represent biological sequences by analyzing the relationships among their elements to capture complex patterns. These methods focus on modeling dependencies between physicochemical properties or sequence positions, enhancing feature extraction for predictive tasks in computational biology. By quantifying correlations, such as auto-covariance for single-property dependencies or cross-covariance for interactions between different properties, these methods provide robust representations that integrate local and partial global sequence information, supporting tasks like RNA classification and epigenetic analysis.

#### 2.3.1. Overview

Correlation-based methods encode biological sequences by quantifying relationships between physicochemical properties across sequence positions. The auto-covariance (AC) [42,43] method measures the correlation of a single property, such as hydrophobicity, between sequence elements separated by a lag, producing vectors with dimensions based on the number of properties (P) and maximum lag (G) (e.g., 50 dimensions for 5 properties and G=10). For nucleotide sequences, this extends to dinucleotide auto-covariance (DAC, k=2) [44] and trinucleotide auto-covariance (TAC, k=3) [45] variants, capturing local and extended patterns. Building on this, the Cross-Covariance auto-covariance (CC) [43,46] method analyzes interactions between different properties, such as polarity and volume, yielding higher-dimensional vectors (P×(P−1)×G), e.g., 200 dimensions for 5 properties and G=10. CC extends to dinucleotide cross-covariance (DCC, k=2) [45] and trinucleotide cross-covariance (TCC, k=3) [45] variants, enhancing multi-property analysis. In addition, the combination of DAC and DCC methods forms dinucleotide auto-cross-covariance (DACC), which encodes vectors with dimensions (P×P×G). The feature encoding length, lower for AC and higher for CC and DACC, balances detailed dependency capture with computational cost, making these methods suitable for tasks like RNA classification and epigenetic analysis. Detailed mathematical formulations are provided in Supplementary Materials (Equations (S8)–(S12)).

#### 2.3.2. Applications

Correlation-based methods, including DAC, TAC, AC, and CC, capture sequence dependencies to advance RNA classification, epigenetic analysis, and sequence analysis with high accuracy. For instance, StackCirRNAPred combined DACC features for predicting long circular RNAs, achieving 83.9% accuracy on a mouse dataset [47]. Similarly, Deep-N6mA employed DCC and TAC features to predict N6-methyladenine sites, achieving 94.23% accuracy, support elucidating regulatory mechanisms of gene expression [48]. Additionally, Uddin et al. used DAC with XGBoost to predict 5-hydroxymethylcytosine modifications, achieving 89.97% accuracy, further supporting epigenetic analysis [49]. An et al. applied DAC, TAC, TCC, and DACC to achieve 83.3% accuracy in DNA barcode classification of poppy species, aiding forensic and ecological applications [50]. These methods, integrated with ML methods like LSTM and XGBoost, deliver robust performance for genomic and epigenetic studies.

#### 2.3.3. Advantages and Limitations

Correlation-based methods offer significant advantages, including the ability to capture intricate sequence dependencies and multi-property interactions, enabling robust performance in RNA classification, epigenetic analysis, and sequence analysis when integrated with machine learning models like deep learning frameworks and ensemble classifiers. However, the high-dimensional feature spaces generated by these methods pose computational challenges, increasing processing time and resource demands for large-scale datasets. Additionally, their performance is sensitive to data quality, as noisy or incomplete datasets may weaken feature robustness.

### 2.4. PSSM-Based Methods

PSSM is a method used to describe the positional specificity of protein sequences. It reflects the probabilities of amino acids appearing at various positions within a protein sequence. This method was initially introduced by Gribskov [51] as a mathematical model for sequence alignment and structural analysis, supporting tasks like protein function prediction and interaction analysis.

#### 2.4.1. Overview

PSSM-based methods encode protein sequences by quantifying the likelihood of amino acid substitutions at each position, reflecting evolutionary patterns. Introduced by Gribskov, the PSSM uses tools like PSI-BLAST [52] to generate an L×20 matrix for a protein sequence of length L, capturing substitution probabilities for each of the 20 amino acids at each position (e.g., a 100 × 20 matrix for a 100-residue sequence). Building on this, in 2001, Chou et al. introduced the Pseudo PSSM (Pse-PSSM) [53], which incorporates sequence-order relationships, producing a 20+20×G vector (e.g., 60 dimensions for G=2) by combining average substitution scores with correlation factors across positional lags. Further advancing this, the k-Tuple Composition PSSM method normalized PSSM scores to extract k-tuple amino acid composition, yielding vectors of $20^{k}$ dimensions (e.g., 20 for k=1 known as amino acid composition PSSM (AAC-PSSM) [54], and 400 for k=2, know as dipeptide PSSM (DPC-PSSM) [54], respectively). PSSM produces feature vector of L×20 for PSSM, 20+20×g for Pse-PSSM, and $20^{k}$ for k-Tuple PSSM, balancing detail and computational cost, suitable for protein function prediction and interaction analysis. Detailed mathematical formulations are provided in Supplementary Materials (Equations (S13)–(S22)).

#### 2.4.2. Applications

PSSM-based methods, including PSSM, k-Tuple Composition PSSM, and Pse-PSSM, leverage evolutionary and sequence information to advance protein function prediction, PPI prediction, and structural analysis with high accuracy across diverse domains. For instance, DeepRank-GNN uses PSSM to predict protein–protein interfaces with 82% accuracy, aiding in the understanding of their interaction mechanisms [55]. PLM-ATG employs k-Tuple Composition PSSM to identify autophagy related proteins with 99.98% AUC, support reveal the coordinated mechanism of autophagy process by autophagy related proteins (ATG) [56]. Ensemble deep learning with PSSM-derived features classifies lipocalin sequences at 97.65% accuracy, support to understand its multiple functions and promotes the development of new disease treatment methods [57]. The prediction method of protein structure categories combining PSSM and PsePSSM features achieved an accuracy of 81.0–89.5% on different datasets, which helps to reveal the recognition of folding patterns [58]. These methods, integrated with advanced machine learning models like graph neural networks, XGBoost, deliver robust performance for genomic and proteomic studies.

#### 2.4.3. Advantages and Limitations

Their primary advantages include the ability to capture intricate evolutionary conservation and sequence-order patterns, providing rich feature representations that enhance predictive accuracy in protein function prediction and PPI prediction. However, the high-dimensional nature of their feature vectors also increases computational complexity, often necessitating dimensionality reduction techniques, such as PCA, to optimize efficiency and scalability in large-scale bioinformatics.

### 2.5. Structure-Based Methods

Structure refers to the local folding and spatiality arrangement of RNA and protein sequences. Specifically, DNA features a double helix with paired nucleotide chains, whereas RNA is more complex [59], exhibiting single-stranded secondary structures (SSs) and various loop formations. Protein structure results from hydrogen bonds between amino acids, which include α-helix, β-sheet, random coil, β-turn, and π-helix. The structure-based methods capture the local folding features of RNA and protein sequences in three-dimensional space and represent these features in a format that ML methods can comprehend, thereby providing a novel perspective for sequence analysis, supporting tasks like RNA and protein function prediction. Given the relatively simple secondary structure of DNA, structure-based methods are primarily applied to RNA and protein. It is essential to note that these structural features are typically predicted using specialized tools, such as RNAFold [60] and Mfold [61]. RNA SSs are represented in dot-bracket format [60,62] and connectivity table (CT) format [63], which provide a concise numerical representation of base positions and pairings.

#### 2.5.1. Overview

Structure-based methods encode RNA and protein sequences by modeling their secondary structure patterns, primarily for RNA and proteins due to DNA’s simple double-helix structure. Introduced by Xue et al. [64], the triplet structure (TS) method represents RNA sequences as 32-dimensional vectors, combining sequence and structural information for trinucleotides (4 nucleotides × 8 paired/unpaired states, e.g., “A(((“ or “U(..”). Su [65] proposed the split protein secondary structure composition (SPSSC) method, which uses tools like RaptorX to predict three-state secondary structure (helix, sheet, coil) [66] and divides protein sequences into m subsequences, each represented by k-tuple frequencies (e.g., m×9 dimensions for k=2). Further advancing this, Liu et al. [67] introduced the Pseudo Structure Status Composition (PseSSC) method, which combines RNA secondary structure states (10 states: 4 unpaired, 6 paired) with sequence-order correlations, yielding a $10^{k}+\lambda$-dimensional vector (e.g., 100+λ for k=2). Structure-based methods produce feature vectors of 32 dimensions for TS, $m\times 3^{k}$ for SPSSC, and $10^{k}+\lambda$ for PseSSC, balancing detail and computational cost, suitable for RNA modification prediction and protein function analysis. Detailed mathematical formulations are provided in Supplementary Materials (Equations (S23)–(S29)).

#### 2.5.2. Applications

Structure-based methods effectively capture structural and sequence patterns for RNA modification prediction, protein function prediction and RNA–protein interaction (RPI) prediction. For instance, AutoBioSeqpy employs TS to predict N7-methylguanosine (m7G) sites with 92.6% accuracy, helping reveal RNA modification mechanisms [68]. XGEM employs TS to identify miRNA precursors with 90.0% accuracy, aiding gene regulation studies [69]. PseSSC enhances pre-miRNA prediction with 85.76% accuracy and RPI prediction with 97.8% accuracy, clarifying the important role of structural features in model representation [67,70]. Integrated with ML methods, these methods advance genomics and proteomic studies.

#### 2.5.3. Advantages and Limitations

Structure-based methods capture intricate local structural and sequence patterns, particularly for RNA and protein sequences. These methods generate biologically meaningful feature representations that reflect secondary structure motifs, such as RNA base pairings or protein helix and sheet configurations, enhancing compatibility with machine learning frameworks like deep neural networks and ensemble classifiers [64,65]. This enables precise modeling of RNA and protein sequences critical for RNA modification prediction and protein function analysis. However, their focus on local structural features limits their ability to capture global sequence contexts, potentially reducing effectiveness in tasks requiring long-range dependencies. Additionally, high-dimensional outputs, especially for TS and PseSSC, increase computational demands, particularly for large-scale datasets. These methods also rely heavily on accurate structural prediction tools, such as RNAFold or RaptorX, and their performance may be compromised by noisy or incomplete structural data [60,66].

## 3. Word Embedding-Based Methods

Computational-based methods, while effective for local patterns, are limited by high dimensionality and local focus. To address these limitations, word embedding-based methods were developed to capture contextual relationships using neural networks. These methods, originally developed in natural language processing (NLP), are driven by advances in ML, particularly deep neural networks, have emerged as powerful tools for biological-sequence representation by mapping sequences to continuous vector spaces that capture semantic and contextual relationships [71,72]. These methods treat biological sequences as language, representing k-mers or residues as “words” and sequences as “sentences.” By training neural networks on large-scale biological datasets, they generate dense, low-dimensional vectors that encode meaningful features, facilitating tasks such as sequence classification, function prediction, and cross-species analysis. These methods are summarized in Table 2.

### 3.1. Local Feature Embedding-Based Methods

These methods focus on local feature extraction at the k-mer or sub-k-mer level, suitable for capturing local patterns and contextual relationships, and are applicable to tasks such as sequence analysis and protein function prediction. They perform well in fine-grained analysis, but struggle to capture long-range dependencies.

#### 3.1.1. Overview

Local feature embedding-based methods encode biological sequences by mapping k-mers into continuous vector spaces using neural network-based methods. Introduced in 2013 by Mikolov et al. [73], Word2Vec uses shallow neural networks with two models: Skip-gram, which predicts context k-mers given a target k-mer, and Continuous Bag-of-Words (CBOW), which predicts a target k-mer from its context. By treating sequences as “sentences” and k-mers as “words,” it generates fixed-length vectors (e.g., 100 dimensions) that capture local sequence patterns. Building on this, in 2014, Asgari and Mofrad proposed BioVec [74], tailored for biological sequences. BioVec includes ProtVec, which segments protein sequences into non-overlapping 3-mers and trains a Skip-gram model on the Swiss-Prot database to produce 100-dimensional embeddings encoding sequence patterns and BioVec-specific physicochemical properties, and GeneVec, which applies a similar method to nucleotide sequences. Further advancing this, Ng et al. introduced DNA2Vec [75], which extends Word2Vec to handle variable-length k-mers (e.g., 3 to 8 nucleotides) in DNA sequences. Using a Skip-gram model trained on large genomic datasets, DNA2Vec generates fixed-length vectors (e.g., 100 dimensions) that capture sequence motifs and contextual relationships. These methods produce configurable fixed-length vectors, typically 100 dimensions, ensuring robustness to sequence length variability and computational efficiency for tasks like sequence analysis and protein function prediction.

#### 3.1.2. Applications

Local feature embedding methods by mapping k-mers or residues into continuous vector spaces, capturing local sequence patterns and contextual relationships. These methods excel in sequence analysis, protein function prediction, and binding site prediction. Word2Vec, via Skip-gram models, classifies protein sequences with 81.14% accuracy, Helping promote the recognition of snake venom proteins and the development of related drugs [76]. BioVec, including ProtVec and GeneVec, can be used for functional classification, structural prediction, and PPI prediction of protein families [74,77]. DNA2Vec identifies transcription factor (TF) binding sites with 83.10% accuracy, supporting gene regulation studies [78]. Integrated with deep learning, these methods enhance genomic and proteomic analysis.

#### 3.1.3. Advantages and Limitations

The key advantages of these methods include their ability to automatically learn features from sequence data, eliminating the need for manually defined features common in earlier computational-based methods, and their production of low-dimensional, fixed-length embeddings that ensure robustness to sequence length variability and improve computational efficiency for tasks like sequence analysis and protein function prediction. These embeddings integrate effectively with ML methods, such as support vector machines and deep neural networks, to achieve robust predictive performance. However, these methods have notable limitations. Their effectiveness relies heavily on large, high-quality training datasets, with smaller or noisy datasets leading to suboptimal embeddings [74]. Additionally, the embeddings, derived from local k-mer patterns, are challenging to interpret biologically, as they do not directly correspond to specific functional or structural properties, limiting their use in tasks requiring mechanistic insights. Furthermore, their focus on local patterns hinders their ability to capture long-range dependencies, a challenge addressed by later methods.

### 3.2. Global Feature Embedding-Based Methods

Global feature embedding-based methods transform biological sequences into continuous vector representations by modeling statistical relationships across entire sequences or datasets, capturing holistic context for tasks like sequence analysis and protein and RNA function prediction. These methods leverage fixed-length encodings, typically in low-dimensional spaces, to balance comprehensive pattern capture with computational efficiency.

#### 3.2.1. Overview

Global feature embedding-based methods encode nucleotide and protein sequences by capturing statistical relationships across entire sequences using neural network-based or matrix factorization methods. Introduced in 2014 by Le and Mikolov as an extension of Word2Vec, Doc2Vec [73,79] treats sequences as “documents” and k-mers as “words,” generating fixed-length vectors (e.g., 100–300 dimensions). It employs two architectures: Distributed Memory (DM), which integrates a document vector with k-mer vectors to predict target words based on sequence context, and Distributed Bag-of-Words (DBOW), which uses the document vector alone to predict sequence k-mers, capturing sequence-wide patterns. Also proposed in 2014 by Pennington et al., Global Vectors (GloVe) [80] builds a co-occurrence matrix from k-mers within a fixed context window (e.g., 5–15 units), counting pairwise co-occurrences across large biological datasets. A logarithmic weighting function reduces the impact of frequent pairs, and weighted least squares optimization factorizes the matrix to produce fixed-length vectors (e.g., 50–300 dimensions). These methods produce fixed-length vectors, typically 100–300 dimensions for Doc2Vec and GloVe, ensuring robustness to sequence length variability and computational efficiency for tasks like sequence analysis and protein and RNA function prediction.

#### 3.2.2. Applications

Global embedding-based methods, including Doc2Vec and GloVe, capture contextual sequence features for precise sequence analysis, protein and RNA function prediction, and PPI prediction. Doc2Vec predicts PPI in human herpesvirus and cross-species datasets with high accuracy, revealing interaction networks [81,82]. It also predicted the circRNA–RNA-Binding Protein (RBP) interaction site with an average AUC of 90.76%, supporting research on circRNA as a biomarker for diagnosing various diseases [83]. GloVe predicts RNA 5-methyluridine (m5U) sites and DNA-binding proteins (DBP) with strong performance, supporting epigenetic analysis [84,85].

#### 3.2.3. Advantages and Limitations

Their primary advantages of global feature embedding-based methods include their ability to capture global sequence patterns, providing a comprehensive representation of sequence context that is more robust to sequence length variability compared to traditional computational-based methods. The low-dimensional embeddings generated by Doc2Vec and GloVe enhance computational efficiency, while their reliance on statistical relationships enables adaptability to sequence analysis and protein and RNA function prediction. However, these methods have notable limitations. These methods depend on large, high-quality datasets to build effective co-occurrence matrices or document vectors, with performance declining for smaller or noisy datasets. Their emphasis on global sequence patterns often sacrifices local details, reducing suitability for fine-grained tasks like motif discovery, which local embedding methods handle more effectively. Moreover, the embeddings’ abstract nature complicates mapping to specific biological properties, such as functional motifs or interaction sites, hindering mechanistic understanding.

## 4. Large Language Model-Based Methods

With the advancement of sequencing technology, feature models of biological data have evolved, leading to the emergence of integrated multi task learning and transfer learning technology models that adapt to constantly changing computing tasks, including sequence analysis, protein structure prediction, and drug discovery. A significant advancement is Protein Language Models (PLMs) [86], which leverage Transformer architectures to model protein sequences with unprecedented accuracy [87]. PLMs are advanced computational methods that apply natural language processing techniques to represent protein sequences as language-like data, using attention mechanisms to capture contextual relationships and long-range dependencies [87]. The attention mechanism, a cornerstone of Transformers, enables PLMs to weigh the importance of different residues in a sequence, effectively modeling long-range dependencies and evolutionary patterns [87]. These methods are summarized in Table 3.

### 4.1. Self-Supervised Learning Methods

Self-supervised learning (SSL) methods are pre-trained through self-supervised tasks, utilizing masked language modeling (MLM) technology to capture context and long-range dependencies in biological sequences for tasks like sequence analysis and protein and RNA structure prediction. In MLM, 15% of sequence tokens (e.g., nucleotides or amino acids) are masked (80% [MASK], 10% random, 10% unchanged) and predicted via cross-entropy loss, enabling models to learn intrinsic patterns from large, unlabeled datasets (e.g., RNAcentral database, UniProt).

#### 4.1.1. Overview

Self-supervised learning methods encode nucleotide and protein sequences using Transformer-based architectures trained on large datasets to capture contextual and long-range dependencies. Protein-focused models emerged with Evolutionary Scale Modeling (ESM) series, notable ESM1b [88] proposed by Rives et al. in 2021., utilizing 33 Transformer encoder layers with 12 attention heads and scaled dot-product attention. Trained on 250 million UniRef50 database protein sequences, ESM1b applies MLM to predict masked amino acids, producing 1280-dimensional embeddings. Elnaggar et al. proposed ProtTrans [89], a family of BERT-based models, includes ProBERT, which utilizes 30 Transformer encoder layers and 16 attention heads. Pre-trained on UniRef100 with 15% MLM, tokenizes sequences into 20 standard amino acids plus special tokens, standardized to 512 residues, yielding 1024-dimensional embeddings. In 2024, Shen et al. introduced RNA-FM [90], which employs 12 Transformer encoder layers with 20 multi-heads self-attention to model RNA sequences. RNA-FM is trained on 23.7 million non-coding RNA sequences from RNAcentral, with T replaced by U. It applies MLM to predict 15% randomly masked nucleotides, generating 640-dimensional embeddings via mean pooling. Subsequently, in 2025, ProLLama [91] extended protein modeling by adapting the LLAMA-2-7B architecture, trained on UniRef50 using autoregressive modeling and leveraging residual connections, layer normalization, and low-rank adaptation to generate embeddings optimized for multi-task protein language processing, including sequence generation and superfamily prediction.

#### 4.1.2. Applications

Self-supervised learning (SSL) methods mark a transformative advancement in biological-sequence representation, capturing contextual. ESM1b supports protein function prediction and binding site identification by encoding evolutionary and contextual patterns, achieving high accuracy in diverse protein analysis tasks [88,92]. ProtBERT enhances protein function and domain classification, as well as binding site identification, by modeling complex residue interactions [89]. RNA-FM excels in RNA 3D structure prediction and classification, leveraging large-scale unlabeled data, enhances performance in scenarios with limited labeled data [90]. Similarly, ProLLama strengthens protein structure prediction and interaction analysis, capturing intricate sequence relationships [91]. These methods integrate seamlessly with machine learning frameworks, leveraging large, unlabeled datasets to deliver robust performance across sequence analysis, protein and RNA structure prediction, and binding site prediction.

#### 4.1.3. Advantages and Limitations

Their primary advantages include their ability to model contextual relationships through attention mechanisms, surpassing the local focus of word embedding-based methods, and their generalizability across sequence analysis and protein and RNA structure prediction due to pre-training on expansive datasets like UniProt and RNAcentral. The high-dimensional, contextualized embeddings provide rich feature representations, adaptable through optional fine-tuning for specific applications, and their data-driven method reduces reliance on predefined features compared to computational-based methods. However, these methods face significant challenges. Their high computational costs, driven by large-scale Transformer architectures and extensive training datasets, restrict accessibility and scalability in resource-constrained settings [59,89,91]. Additionally, their complex, high-dimensional embeddings obscure direct connections to biological properties, such as secondary structures or binding affinities, limiting mechanistic insights and interpretability. Performance is heavily dependent on large, high-quality datasets, with smaller or noisy datasets risking suboptimal embeddings.

### 4.2. Multi-Task Learning Methods

Multi-task learning (MTL) methods integrate multiple pre-training objectives, such as sequence analysis, structure prediction, and functional annotation, to generate universal embeddings for biological sequences. Unlike SSL, which focuses on single-task pre-training like MLM, MTL jointly optimizes diverse tasks [93] (e.g., MLM, contact map prediction, knowledge embedding) using composite loss functions (e.g., weighted sums of task-specific losses). MTL models, typically based on Transformer architectures, leverage large, annotated datasets (e.g., UniProt, ProteinKG25) to encode sequence, structural, and functional relationships. This multi-objective method increases training complexity but produces versatile embeddings suited for diverse biomolecular tasks, contrasting with SSL’s task-agnostic, sequence-focused embeddings.

#### 4.2.1. Overview

Multi-task learning methods encode nucleotide and protein sequences using Transformer-based architectures trained on large datasets to model sequence, structural, and functional properties. Earlier, protein-focused models emerged with Tasks Assessing Protein Embeddings (TAPE) in 2019 by Rao et al. [93], using 12 Transformer encoder layers with 8 self-attentions heads to tokenize protein sequences into 20 standard amino acids plus special tokens. TAPE optimizes multiple tasks—secondary structure prediction, contact map prediction, and remote homology detection on independent data sources such as Pfam database, CB513 and ProteinNet. In 2022, Zhang et al. proposed OntoProtein [94], combining MLM with knowledge graph embedding using ProteinKG25 (612,483 entities, 4,990,097 triplets). A hybrid encoder, combining ProtBERT-based protein encoding with BERT-based Gene Ontology (GO) term encoding, employs contrastive learning to align the semantic association between proteins and GO to generate embeddings. In 2024, RNAErnie [95] employs 12 Transformer encoder layers with multi-head self-attention to process RNA sequences. Pre-trained on 23.7 million non-coding RNA sequences from RNAcentral database, RNAErnie uses a motif-aware MLM strategy, masking 15% of nucleotides (80% [MASK], 10% random, 10% unchanged) at base, subsequence, and motif levels to predict structural motifs and sequence context, generating 768-dimensional embeddings via mean pooling. It also employs type-guided fine-tuning, predicting RNA types (e.g., microRNA (miRNA), long non-coding RNA (lncRNA)) to enhance adaptability, with flexible fine-tuning architectures (FBTH, TBTH, STACK). Subsequently, in 2024, Abramson et al. introduced AlphaFold3 [22], a diffusion-based model integrating Transformer-based sequence encoding to predict protein, small molecule, and nucleic acid interactions. Trained on PDB and chemical databases, it generates embeddings for biomolecular complexes. Similarly, Krishna et al. proposed RoseTTAFold All-Atom [23] in 2024, a Transformer-based model trained on PDB and UniProt for protein-ligand and protein-nucleic acid interactions, using multi-task objectives for structure prediction and functional annotation. In 2025, ESM3 [96] introduced multimodal generative modeling, encoding sequences, structures, and functions as discrete tokens in a 98-billion-parameter Transformer. ESM3, trained on 3.15 billion sequences, 236 million structures, and 539 million annotations from curated datasets (e.g., UniProt, PDB), and uses generative MLM to predict masked tokens across modalities, producing high-dimensional embeddings [96]. These methods produce feature encodings, ranging from 256 to 1024 dimensions, balancing comprehensive multi-modal representation with computational efficiency for tasks like sequence analysis, structure prediction, and functional annotation.

#### 4.2.2. Applications

Multi-task learning (MTL) methods capturing sequence, structural, and functional properties for sequence analysis, structure prediction, and functional annotation. TAPE supports protein function prediction and structural analysis, effectively modeling complex protein characteristics [93]. OntoProtein enhances protein function prediction and annotation by integrating biological knowledge with sequence data, improving accuracy in functional analysis [94]. RNAErnie excels in RNA structure prediction and type classification, such as identifying miRNA and lncRNA, by enhancing structural and functional insights [95]. AlphaFold3 predicts protein-small molecule, protein-nucleic acid interactions and structure prediction, aiding drug discovery [22]. RoseTTAFold All-Atom supports protein-ligand binding and functional genomics [23]. ESM3 enables protein structure generation, function prediction, and cross-modal reasoning, offering exceptional scalability across diverse computational biology tasks [96]. These methods integrate seamlessly with machine learning frameworks, leveraging diverse data to deliver robust performance in sequence analysis, structure prediction, and functional annotation.

#### 4.2.3. Advantages and Limitations

Their primary advantages include their ability to jointly model sequence, structural, and functional properties, yielding versatile embeddings that outperform single-task SSL methods in sequence analysis, structure prediction, and functional annotation. The incorporation of external knowledge (e.g., GO terms in OntoProtein) and multimodal data (e.g., ESM3) enhances generalizability, while fine-tuning strategies improve adaptability to specific tasks. However, MTL methods face significant challenges. Their high computational complexity, stemming from multi-objective optimization and large-scale datasets, requires substantial resources, limiting accessibility [22,23,89,94,96]. Moreover, the multifaceted embeddings blur direct associations with specific biological features, such as binding sites or functional motifs, limiting mechanistic interpretation and interpretability. Performance heavily depends on large, high-quality annotated datasets, with noisy or limited data degrading embedding quality, a challenge shared with SSL methods.

## 5. Challenges and Future Directions

The development of biological-sequence representation methods, from computational-based methods to word embedding-based methods and LLM-based methods, has advanced tasks like sequence analysis, structure prediction, functional annotation, and interaction prediction. Each stage, however, faces specific challenges tied to feature encoding length and computational demands, guiding targeted improvements for computational biology applications.

Computational-based structure methods, such as TS and PseSSC, encode RNA and protein sequences into high-dimensional vectors (e.g., 64 dimensions for TS, $10^{k}+\lambda$ for PseSSC) to capture local structural patterns like RNA base pairings or protein secondary structures. These high-dimensional outputs increase computational complexity for tasks like N7-methylguanosine site prediction and pre-miRNA identification, while their focus on local features often misses global sequence context, limiting performance in tasks requiring long-range dependencies [67,68]. Moving to word-embedding methods, local feature embeddings (Section 3.1), like Word2Vec and DNA2Vec, generate fixed-length 100-dimensional vectors using shallow neural networks. Their reliance on extensive training data leads to suboptimal embeddings for smaller or noisy datasets, impacting transcription factor binding prediction [74,75]. Global feature embeddings (Section 3.2), such as Doc2Vec and GloVe, produce 100–300-dimensional vectors by modeling sequence-wide statistical patterns but often overlook local motifs, reducing effectiveness in RNA methylation site prediction [83,84]. Progressing to LLM-based methods, self-supervised learning methods (Section 4.1), like RNA-FM and ESM1b, use Transformer architectures to generate high-dimensional embeddings. These models, trained on large datasets like RNAcentral database and UniProt, demand significant computational resources for RNA 3D structure prediction and protein function annotation [88,90]. Multi-task learning methods (Section 4.2), such as RNAErnie and ESM3, integrate objectives like MLM and knowledge embedding, producing 256–1024-dimensional embeddings. Their complex architectures and dependence on annotated datasets like ProteinKG25 increase computational costs and sensitivity to data quality, affecting tasks like RNA classification, RNA-RNA interaction, and protein structure prediction.

Common challenges include data imbalances (e.g., under-represented RNA types in RNAcentral database), sequencing errors introducing noise, and overfitting risks in high-dimensional embeddings due to limited labeled data. Specially, the complexity of computation is a noteworthy issue as it brings about the scarcity of computing resources. In current research, strategies such as sparse attention mechanisms [97] are used to reduce memory and time requirements by focusing on key sequence interactions, while model compression techniques such as knowledge distillation and low-rank adaptation [98,99] have created lightweight models with comparable performance. Mixed precision training and recent combinations of k-mer features with small language models have further improved accessibility for resource-constrained researchers, as demonstrated by recent models for plant genome annotation and prediction of regulatory element strength [100]. These optimization strategies alleviate the high resource requirements of LLM, enabling it to be deployed on standard hardware for tasks such as sequence analysis and functional annotation, directly addressing the accessibility challenges of resource-constrained researchers.

However, another noteworthy challenge is that the high-dimensional embeddings generated by LLMs limit their ability to generate substantial biological insights due to interpretability limitations. To address this issue, feature attribution methods such as Shapley additive explanations (SHAP) and local interpretable model-agnostic explanations (LIME) can identify key sequence features that are helpful for prediction, as shown in protein functional annotation [101,102]. Visualization techniques such as t-Distributed Stochastic Neighbor Embedding (t-SNE) and Uniform Manifold Approximation and Projection (UMAP) map high-dimensional embeddings to low-dimensional spaces for functional annotation and interaction prediction, as well as gene function prediction [103]. Integrating biological priors, such as Gene Ontology (GO) terminology or known pathways, can enhance understanding of mechanisms, such as OntoProtein for protein function annotation. In addition, analyzing Transformer attention weights, such as RNAErnie for RNA structure prediction, highlights the key sequence regions that drive prediction [95]. These methods collectively bridge the gap between complex LLM outputs and specific biological insights, enhancing their practicality in tasks such as functional annotation and interaction prediction.

To address these challenges, future advancements in biological-sequence representation will focus on unifying the strengths of computational, word embedding, and LLM-based methods to enhance their applicability across sequence analysis, structure prediction, functional annotation, and interaction prediction. Integrating multimodal data, such as sequences, secondary structures, and functional annotations from datasets like ProteinKG25, will enable richer representations for tasks like interaction prediction and sequence analysis. For instance, combining single-cell RNA sequencing (scRNA-seq) and Assay for Transposase-Accessible Chromatin sequencing (ATAC-seq) data with cross-modal generative models like ESM3 can capture complex biological patterns, improving the accuracy of function annotation and drug discovery, and combining sequence-order correlations with structural features, as seen in PseSSC, and functional annotations, as in OntoProtein, can better model complex biological interactions [67,94]. Enhancing interpretability by mapping high-dimensional embeddings to biological properties, such as binding affinities for RNA methylation sites or structural motifs for protein functions, will improve mechanistic insights for functional annotation and interaction prediction. Emerging explainable AI (XAI) techniques, such as SHAP and attention visualization, along with knowledge graph-based embeddings like OntoProtein, can elucidate feature contributions, bridging the gap between embeddings and biological mechanisms [101]. Optimizing computational efficiency through techniques like sparse attention mechanisms (e.g., Linformer and Performer) and model compression methods (e.g., knowledge distillation, quantization) can significantly lower the computational burden, enabling scalability on standard hardware [87]. Additionally, addressing data imbalances through synthetic data generation and cross-species transfer learning will enhance model robustness for tasks like non-coding RNA classification. Developing robust denoising techniques to mitigate sequencing errors will further improve performance in tasks like interaction prediction [104,105]. Optimizing feature encoding length, such as balancing the high-dimensional outputs of PseSSC and ESM with low-dimensional embeddings like Word2Vec through dimensionality reduction, will ensure a trade-off between capturing complex patterns and computational tractability. These advancements will drive the next phase of computational biology, enabling more accurate and interpretable sequence analysis.

## 6. Conclusions

This paper systematically reviews biological-sequence representation methods, tracing their development through computational-based methods, word embedding-based methods, and LLM-based methods, which collectively underpin the extraction of biological insights from sequence data. Computational-based methods, such as k-mer, PSSM, and structure-based methods like TS and SPSSC, excel in capturing local patterns and structural features, making them ideal for sequence analysis and structure prediction, though limited by high-dimensionality and local focus [28]. Word embedding-based methods, including Word2Vec, BioVec, and Doc2Vec, marked a shift toward contextual feature learning, enhancing performance in applications like transcription factor binding prediction and protein–protein interaction analysis, but constrained by data requirements and interpretability challenges [88,94]. LLM-based methods, such as ESM3, ProtBERT, and ProLLama, leverage attention mechanisms to model long-range dependencies and evolutionary patterns, achieving state-of-the-art results in structure prediction, functional annotation, and drug discovery, despite high computational costs [59,91]. While computational-based methods dominate in efficiency and quantity, structure-based methods are increasingly recognized for their ability to reflect the intricate interplay between sequence, structure, and function. Emerging trends in representation learning and multi-source, multimodal data integration are enhancing feature extraction, with studies exploring these to improve robustness [106,107]. Although LLM-based methods are still developing, they demonstrate significant potential to transform computational biology. Future progress will hinge on addressing computational, data, and interpretability challenges, ensuring these methods continue to evolve and drive transformative discoveries in biological research.

## Figures and Tables

**Table 1:** Summary of computational-based methods.

Methods	Core Applications	Advantages	Limitations
k-mer-based	Genome assembly, motif discovery, sequence classification	Computationally efficient, captures local patterns	High dimensionality, limited long-range dependency capture
Group-based	Protein function prediction, Protein annotation, protein–protein interaction prediction	Encodes physicochemical properties, biologically interpretable	Sparsity in long sequences, parameter optimization needed
Correlation-based	RNA classification, epigenetic modification prediction	Models complex dependencies, robust for multi-property interactions	High computational cost, limited for RNA trinucleotide correlations
PSSM-based	Protein structure/function prediction, PPI prediction	Leverages evolutionary conservation, robust feature extraction	Dependent on alignment quality, computationally intensive
Structure-based	RNA modification prediction, protein function prediction	Captures local structural motifs, biologically meaningful	Relies on accurate structural predictions, limited global context

Table 1 shows a summary of computation-based methods. These methods have some common advantages: all methods are effectively integrated with machine learning models (such as SVM, RF, deep neural network and extreme gradient boosting (XGBoost)), improving the prediction performance across tasks. There are also some common limitations: high-dimensional output is a challenge for most methods, and dimensionality reduction techniques (such as principal component analysis (PCA)) are usually required for efficient processing.

**Table 2:** Summary of word embedding-based methods.

Methods	Core Applications	Advantages	Limitations
Local feature embedding-based	Protein sequence classification, transcription factor binding prediction, gene annotation	Captures short-range patterns, robust to sequence length variability, computationally efficient	Limited to local dependencies, requires large training datasets, lacks direct biological interpretability
Global feature embedding-based	Protein function prediction, RNA methylation site prediction, regulatory RNA identification	Models sequence-wide context, adaptable to diverse tasks, robust to variable sequence lengths	Misses fine-grained local patterns, computationally intensive, sensitive to dataset quality

Table 2 shows a summary of word embedding-based methods. These methods have some common advantages: All methods integrate effectively with machine learning models (e.g., SVM, deep neural networks, ensemble classifiers), enhancing predictive performance across computational biology tasks. Common Limitation: Embeddings lack direct interpretability, as they are not explicitly tied to biological features (e.g., binding affinities, structural motifs), limiting mechanistic insights. Performance depends on large, high-quality datasets, with smaller or noisy datasets risking suboptimal embeddings.

**Table 3:** Summary of large language model-based methods.

Methods	Core Applications	Advantages	Limitations
Self-supervised learning	RNA/protein structure prediction, binding site identification, functional annotation	Models complex sequence relationships, generalizable with unlabeled data, high predictive accuracy	High computational cost, requires large datasets
Multi-task learning	RNA type/structure classification, protein function/structure prediction, cross-modal analysis	Integrates sequence, structure, and function data, adaptable to diverse tasks, enhanced by multi-modal learning	Complex training process, resource-intensive, sensitive to annotated dataset quality

Table 3 shows a summary of word embedding-based methods. These methods have some common advantages: All methods integrate effectively with machine learning frameworks (e.g., deep neural networks, ensemble classifiers), enhancing predictive performance in computational biology tasks. Common Limitation: Embeddings lack direct interpretability, as they are not explicitly tied to biological features (e.g., binding affinities, structural motifs). Performance relies on large, high-quality datasets, with smaller or noisy datasets risking suboptimal results.

## Data Availability

Not applicable.

## Supplementary Materials

The following supporting information can be downloaded at: https://www.mdpi.com/article/10.3390/biology14091137/s1, Table S1: Amino acids grouping in the CTD method; Figure S1: The detail of the definition and description for CT method; Equations (S1)–(S4): The detailed mathematical formulations for the k-mer-based methods; Equations (S5)–(S7): The detailed mathematical formulations for the group-based methods; Equations (S8)–(S12): The detailed mathematical formulations for the correlation-based methods; Equations (S13)–(S22): The detailed mathematical formulations for the PSSM-based methods; Equations (S23)–(S29): The detailed mathematical formulations for the structure-based methods.

## Abbreviations

The following abbreviations are used in this manuscript:

ML	Machine Learning
SVM	Support Vector Machine
RF	Random Forest
NN	Neural Network
LR	Linear Regression
AUC-ROC	Area Under the Receiver Operating Characteristic Curve
CNN	Convolutional Neural Network
LSTM	Long Short-Term Memory
LLM	Large Language Model
PPI	Protein–Protein Interaction
XGBOOST	eXtreme Gradient Boosting
PCA	Principal Component Analysis
PSSM	Position Specific Scoring Matrix
MNC	Mononucleotide Composition
DNC	Dinucleotide Composition
TNC	Trinucleotide Composition
AAC	Amino Acid Composition
DPC	Dipeptide Composition
TPC	Tripeptide Composition
CTD	Composition, Transition, and Distribution
CT	Conjoint Triad
AC	Auto-Covariance
CC	Cross-Covariance
DAC	Dinucleotide Auto-Covariance
TAC	Trinucleotide Auto-Covariance
DCC	Dinucleotide Cross-Covariance
TCC	Trinucleotide Cross-Covariance
DACC	Dinucleotide Auto- and Cross-Covariance
Pse-PSSM	Pseudo Position Specific Scoring Matrix
SPSSC	Split Protein Secondary Structure Composition
TS	Triplet Structure
MLM	Masked Language Modeling
PLM	Protein Language Model
GO	Gene Ontology
TAPE	Tasks Assessing Protein Embeddings
SHAP	SHapley Additive exPlanations
LIME	Local Interpretable Model-agnostic Explanations
