# Supplementary material for: Biological Sequence Representation Methods and Recent Advances: A Review
Source: Biology (Basel). 2025 Aug 27;14(9):1137. doi: 10.3390/biology14091137 (PMC12467266; doi:10.3390/biology14091137)
Supplement: Supplementary file 1 [file biology-14-01137-s001.zip › biology-3788189-supplementary.pdf]

Supplementary Materials

S1.1 K-mer-Based Methods

This section provides detailed mathematical formulations for the k-mer based methods discussed in manuscript Section 2.1, including basic k-mer and gapped k-mer methods.

S1.1.1 Basic k-mer

The fundamental form of the basic k-mer method encodes a sequence as a vector of length Σ^k-dimension, which can be defined as Equation. (S1).

$$\mathbf{V_{k-mer}} = [\mathbf{f_1^{(k)}}, \mathbf{f_2^{(k)}}, \cdots, \mathbf{f_{|\Sigma|^k}^{(k)}}] \tag{S1}$$

Where $f_i^{(k)}$ denotes the frequency of k-mers that appear in the sequence, as given by Equation. (S2).

$$\mathbf{f_i^{(k)}} = \frac{\mathbf{N_i}}{\mathbf{L - k + 1}} \tag{S2}$$

Here, L deotes the length of sequence, $L - k + 1$ denotes the count of overlapping k-mers, and N_i denotes the count of occurrences of the i-th k-mer in the sequence. The vector dimension is $|\Sigma|^k$, where Σ denotes the sequence alphabet ($|\Sigma| = 4$ for nucleotide sequences, $|\Sigma| = 20$ for protein sequences).

S1.1.2 Gapped k-mer

This method is a variant of the k-mer method that incorporates gaps, which can be described as follows: let M denote the total number of gapped k-mers. This method relies on the two parameters l and m, l denotes the total length of the subsequences including gaps, while m denotes the number of non-gapped positions in each subsequence. Therefore, the length of the gap is $l - m$. The sequence can be represented as a vector, as shown in Equation. (S3).

$$\mathbf{V_{Gapped\ k-mer}} = [\mathbf{f_1}, \mathbf{f_2}, \cdots, \mathbf{f_M}] \tag{S3}$$

Here, f_i denotes the count of the i-th gapped k-mer, while $M = \binom{l}{m} \cdot |\Sigma|^m$ denotes the total number of gapped k-mers. In order to solve the problem of data sparsity caused by the increase of k value, a new kernel function called gkm kernel was introduced [46], which measures the similarity between two sequences by calculating the inner product of all possible gapped k-mers between them. Specifically, the calculation formula for the kernel function is as Equation (S4):

$$\mathbf{K(S_1, S_2)} = \frac{< \mathbf{V_{S_1}}, \mathbf{V_{S_2}} >}{||\mathbf{V_{S_1}}||\ ||\mathbf{V_{S_2}}||} \tag{S4}$$

Here, $< V_{S_1}, V_{S_2} >= \sum_{i=1}^{M}(f_i^{S_1}, f_i^{S_2})$ denotes the product of feature vector V_{S_1} and V_{S_2}. $||V_S|| = \sqrt{< V^S, V^S >}$ denotes the length of the feature vector. In order to furture efficiently calculate kernel functions, the author developed a tree-based data structure. This structure avoids directly calculating all possible gapped k-mer counts by storing all possible l-mers and their occurrences in the training sequence.

S1.2 Group-Based Methods

This section provides detailed mathematical formulations for the group-based methods discussed in manuscript manuscript Section 2.2, including Composition, Transition, and Distribution, and Conjoint Triad methods

S1.2.1 Composition, Transition, and Distribution

The composition, transition (frequencies of group switches, e.g., polar to hydrophobic), and distribution (ratios of group positions, e.g., first, 25%, 50%, 75%, 100%) method (CTD). This method captures general features and patterns, independent of specific structural information and classifies amino acids into three categories: polar (P), neutral (N), and hydrophobic (H), as shown in Table S1.

Table S1 Amino Acids Grouping in the CTD Method

Property	Group 1 (P)	Group 2 (N)	Group 3 (H)
Hydrophobicity	R, K, E, D, Q, N	G, A, S, T, P, H, Y	C, V, L, I, M, F, W
Normalized van der Waals volume	0-2.78<br>G, A, S, C, T, P, D	2.95-4.0<br>N, V, E, Q, I, L	4.43-8.08<br>M, H, K, F, R, Y, W
Polarity	4.9-6.2<br>L, I, F, W, C, M, V, Y	8.0-9.2<br>P, A, T, G, S	10.4-13.0<br>H, Q, R, K, N, E, D
Polarizability	0-0.108<br>G, A, S, D, T	0.128-0.186<br>C, P, N, V, E, Q, I, L	0.219-0.409<br>K, M, H, F, R, Y, W

The vector of CTD can be defined using Equation.(S5):

$$\mathbf{V_{CTD}} = [\mathbf{c_g}, \mathbf{t_{g,h}}, \mathbf{d_{g,p}}] \quad (S5)$$

Where $g \in \{P, N, H\}$ represents one of the three physicochemical groups. $h \in \{P, N, H\} \setminus \{g\}$ denotes the other group in a transition pair. $p \in \{25\%, 50\%, 75\%, 100\%\}$ indicates the positional index for distribution. $c_g = \frac{N_g}{L}$ represents the composition ratio of group (g), $t_{g,h} = \frac{N_{g,h}}{L}$ denotes the transition frequency between groups (g) and (h), $d_{g,p} = \frac{N_{g,p}}{L}$ indicates the distribution ratio of group (g) at position (p). L denotes the sequence length. The resulting CTD vector has a fixed dimension of 21, comprising 3 composition features, 3 transition features, and 15 distribution features (5 positions for each of the 3 groups).

S1.2.2 Conjoint Triad

The conjoint triad (CT) method groups amino acids into seven categories and represents sequences as binary vectors (V, F). Here, V denotes the vector space of sequence features and v_i represents a triad type. F denotes the frequency vector corresponding to V, where the value of the i-th dimension of $F(f_i)$ denotes the frequency of type v_i appearing, as shown in Figure S1.

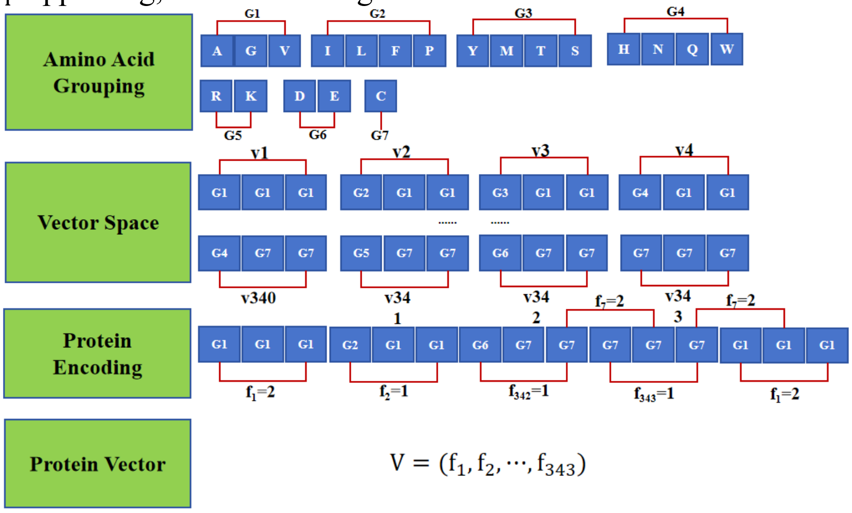

Figure S1. illustrates the detail of the definition and description for (V, F)

This basis for grouping includes polar, non-polar, and charged amino acids, as well as dipoles and side chain volumes. Subsequently, by classifying three consecutive amino acids into one of these categories, a total of $7^3 = 343$ distinct triplets are obtained.

By calculating the frequency of each triad type, a 343-dim vector can be obtained, as depicted in Equation.(S6).

$$\mathbf{V_{CT}} = [\mathbf{f_1}, \mathbf{f_2}, \mathbf{f_3}, \cdots, \mathbf{f_{343}}] \quad (S6)$$

Here, f_t denotes the count of the i-th triad type, as defined in Equation.(S7).

$$\mathbf{f_t = \frac{N_t}{L-2}} \tag{S7}$$

Here, N_t is the number of occurrences of the triad type t in the sequence, and $L-2$ is the total number of consecutive triads.

S1.3 Correlation-Based Methods

This section provides detailed mathematical formulations for the correlation-based methods discussed in manuscript Section 2.3, including auto-covariance (AC) and cross-covariance (CC) methods. These formulas enable precise feature extraction by modeling dependencies between physicochemical properties in biological sequences, supporting applications such as RNA classification and epigenetic modification prediction. For further details on their applications, refer to manuscript Section 2.3.

S1.3.1 Auto Covariance

The auto-covariance method quantifies the correlation of a single physicochemical property between sequence elements separated by a positional lag. It is effective for capturing local and partial global sequence patterns, such as those used in dinucleotide (DAC) and trinucleotide (TAC) variants. The feature vector for AC is defined as: Equation (S8).

$$\mathbf{V_{AC} = [AC(p,g) | p \epsilon P, g = 1, 2, \cdots, G]} \tag{S8}$$

Where, P represents a physicochemical property and G denotes the positional lag. $AC(p, g)$ is the auto-covariance value based on the physicochemical property p of the k-mer element gap g. It can be defined by Equation (S9).

$$\mathbf{AC(p,g) = \frac{1}{L-g}\sum_{i=1}^{L-g}(P_i(p) - \bar{P}(p))(P_{i+g}(p) - \bar{P}(p))} \tag{S9}$$

Where, p_i denotes the k-mer element at position i, p_{i+g} is the k-mer element at position $i+g$. $\bar{P}(p)$ denotes the average value of the current physicochemical properties of p, which is defined by the following Equation (S10).

$$\mathbf{\bar{P}(p) = \frac{\sum_{i=1}^{L-g} P_i(p)}{L}} \tag{S10}$$

For nucleotide sequence, when k=2, the AC method can be derived as dinucleotide auto-covariance (DAC). Similarly, when k=3, the method can be derived as trinucleotide auto-covariance (TAC).

S1.3.2 Cross Covariance

This method can be defined by the following Equation (S11).

$$\mathbf{V_{CC} = [CC(p_1, p_2, g) | p_1, p_2 \epsilon P, p_1 \neq p_2, g = 1, 2, \cdots, G]} \tag{S11}$$

Where, P and G has the same meaning as Eq (S8). $\mathbf{v_{p_1,p_2,g,k}}$ is the cross-covariance value based on the physicochemical property pairs (p_1, p_2) of the k-mer element interval g-spaced. It can be defined by Equation (S12).

$$\mathbf{CC(p_1,p_2,g) = \frac{1}{L-g}\sum_{i=1}^{L-g}(P_i(p_1) - \bar{P}(p_1))(P_{i+g}(p_2) - \bar{P}(p_2))} \tag{S12}$$

Where, P_i and P_{i+g} has the same meaning as in Equation (S9). p_1 and p_2 denote two different physicochemical properties of the current element, and $\overline{P}(p_1)$ and $\overline{P}(p_1)$ denote the average values of the current physicochemical properties p_1 and p_2.

For nucleotide sequence, when $k = 2$, the CC method can be derived as dinucleotide auto-covariance (DCC). Similarly, when $k = 3$, the method can be derived as trinucleotide auto-covariance (TCC).

S1.4 PSSM -Based Method

Position-specific scoring is a method used to describe the positional specificity of biological sequences. It reflects the probabilities of elements appearing at various positions within a protein sequence.

S1.4.1 Position Specific Scoring Matrix

The PSSM encompasses the probability of a specific amino acid occurring within a protein sequence over the course of the species' evolutionary process, which can be defined as shown in Equation. (S13).

$$\mathbf{S_{PSSM}} = \begin{bmatrix} \mathbf{p_{1,1}} & \mathbf{p_{1,2}} & \cdots & \mathbf{p_{1,20}} \\ \mathbf{p_{2,1}} & \mathbf{p_{2,2}} & \cdots & \mathbf{p_{2,20}} \\ \cdots & \cdots & \cdots & \cdots \\ \mathbf{p_{L,1}} & \mathbf{p_{L,2}} & \cdots & \mathbf{p_{L,20}} \end{bmatrix}_{\mathbf{L\times 20}} \tag{S13}$$

Here $i \in \{1,2,\cdots,L\}, j \in \{1,2,\cdots,20\}$, $p_{i,j}$ denotes the probability of being replaced at position (i,j). Therefore, the protein sequence can be defined as shown in Equation. (S14).

$$\mathbf{V_{PSSM}} = [\mathbf{p_{1,j}}, \mathbf{p_{2,j}}, \cdots, \mathbf{p_{L,j}}] \tag{S14}$$

S1.4.2 k-Tuple Composition PSSM

K-tuple-PSSM is a representation method that utilizes normalized PSSM to extract k-tuple amino acid composition features. This method embeds protein sequences into a 20-dimensional vector. Normalize PSSM to a range between 0 and 1 using the following Equation. (S15).

$$\mathbf{P'(i,j)} = \frac{\mathbf{1}}{\mathbf{1 + e^{-P(i,j)}}} \tag{S15}$$

Where $P(i,j)$ is the original PSSM for amino acid. This method generalizes feature as Equation. (S16):

$$\mathbf{V_{K-Tuple-PSSM}} = [\mathbf{f_1}, \mathbf{f_2}, \cdots, \mathbf{f_n}] \tag{S16}$$

Where $n = 20^k$ and:

$$\mathbf{f_i} = \frac{\mathbf{1}}{\mathbf{L - k + 1}} \sum_{\mathbf{m=1}}^{\mathbf{L-k+1}} \prod_{\mathbf{t=1}}^{\mathbf{k}} \mathbf{P'(m + t - 1, a_{i,t})} \tag{S17}$$

Here, $a_{i,t}$ is the t-th amino acid in the i-th k-tuple, and L is the sequence length.

When $k = 1$, the method is derived to amino acid composition PSSM (AAC-PSSM), which can generate a vector of length 20, as shown in the Equation. (S18):

$$\mathbf{V_{AAC-PSSM}} = [\mathbf{f_1}, \mathbf{f_2}, \cdots, \mathbf{f_{20}}] \tag{S18}$$

For $k = 2$, the method is derived to dipeptide composition PSSM (DPC-PSSM), which can generate a vector of 400-dim, as shown in the Eq. (S19):

$$\mathbf{V_{DPC-PSSM}} = [\mathbf{f_{1,1}}, \mathbf{f_{1,20}}, \cdots, \mathbf{f_{2,1}}, \mathbf{f_{2,20}}, \cdots, \mathbf{f_{20,1}}, \cdots, \mathbf{f_{20,20}}] \tag{S19}$$

S1.4.3 Pseudo PSSM

The method employs Eq. (S15) to calculate the average of each column in the normalized original PSSM, yielding a vector of dimensions $20 + 20 \times g$, as depicted in Eq. (S20):

$$\mathbf{V_{PSE-PSSM}} = [\mathbf{f_1}, \mathbf{f_2}, \mathbf{f_3}, \cdots, \mathbf{f_1^g}, \mathbf{f_2^g}, \cdots, \mathbf{f_\lambda^g}] \quad \text{(S20)}$$

Where f_i represents the average score of the amino acid residues in the protein sequence when changed to amino acid type j during the evolution process. This is defined by Equation. (S21):

$$\mathbf{f_i} = \frac{\mathbf{1}}{\mathbf{L}} \sum_{\mathbf{i=1}}^{\mathbf{L}} \mathbf{P_{i,j}} \ (\mathbf{j} = \mathbf{1}, \mathbf{2}, \cdots, \mathbf{20}) \quad \text{(S21)}$$

Here, $P_{i,j}$ is the value of the original PSSM, and f_j^g is expressed as shown in Equation. (S22):

$$\mathbf{f_j^g} = \frac{\mathbf{1}}{\mathbf{L-g}} \sum_{\mathbf{i=1}}^{\mathbf{L-g}} [\mathbf{P_{i,j}} - \mathbf{P_{i+g,j}}]^2 \ (\mathbf{j} = \mathbf{1}, \mathbf{2}, \cdots, \mathbf{20}; \mathbf{g} < \mathbf{L}) \quad \text{(S22)}$$

Where f_j^0 is the correlation factor obtained by coupling the most contiguous PSSM scores along the protein chain for amino acid type j, and f_j^1 is the correlation factor obtained by coupling the second-most contiguous PSSM scores, and so forth.

S1.5 Structure-Based Methods

Structure refers to the local folding and spatiality of these biomolecules. Specifically, DNA features a double helix with paired nucleotide chains, whereas RNA is more complex exhibiting single-stranded structures and various loop formations. Protein structure results from hydrogen bonds between amino acids, which include α-helix, β-sheet, random coil, beta-turn, and π-helix.

1.5.1 Triplet Structure

This method did not consider the pairing direction of the left and right brackets in the nucleotide sequence. Therefore, in the predicted secondary sturcture, each nucleotide can have only two states: paired or unpaired. Any group of three adjacent nucleotides can exhibit $2^3 = 8$ possible structural compositions. Therefore, there will be 4×8=32 possible trinucleotide combinations that contain both structural and sequence information. This method can be defined as a nucleotide sequence of 32-dim feature vector, as shown in Equation (S23).

$$\mathbf{V_{TS}} = [\mathbf{v_1^A}, \mathbf{v_2^A}, \dots, \mathbf{v_{32}^U}] \quad \text{(S23)}$$

Where v_i^A denotes the normalized frequency of three consecutive nucleotides with the middle nucleotide being A.

S1.5.2 Split Protein Secondary Structure Composition

SPSSC first utilizes the RaptorX server to obtain the three-state SS information of the protein, represented by the letters E, H, and C. Subsequently, the SS sequence is divided into m subsequences, with each subsequence represented by the frequency of k-tuple occurrences. When $k = 2$. Each subsequence V_i can be represented as a row of length $3^2 = 9$, as shown in Equation. (S24).

$$\mathbf{V_{SPSSC}} = \left[\mathbf{v_1}, \mathbf{v_2}, \cdots, \mathbf{f_{m \cdot 3^k}}\right] \quad \text{(S24)}$$

Where, $v_i = \frac{N_i}{L-1}$ denotesis the frequency of k-tuples in subsequences, and N_i denotes the number of occurrences of k-tuples. Consequently, the characterized subsequences are fused, enabling the secondary struture sequence of the protein to be converted into an $(m \times 3^k)$-dim vector. Additionally, the parameters m and k in the method significantly influence the results.

S1.5.3 Pseudo Structure Status Composition

The pseudo structural state composition (PseSSC) combines structural and sequence features This method employs the composition of secondary structures to represent nucleotide sequences, as illustrated in Equation. (S25).

$$\mathbf{R} = (\boldsymbol{\Psi}_1, \boldsymbol{\Psi}_2, \boldsymbol{\Psi}_3, \boldsymbol{\Psi}_4, \cdots, \boldsymbol{\Psi}_L) \tag{S25}$$

Here, Ψ_i denotes any of the ten structure states, $\Psi_i \in \{A, C, G, U, A-U, U-A, G-C, C-G, G-U, U-G\}$ for $i = 1, 2, 3, \cdots, L$. Among them, A, C, G, and U represent the structural states of four unpaired bases, while $A-U, U-A, G-C, C-G, G-U$, and $U-G$ denote the structures of six paired bases. This method can be defined as a nucleotide sequence of $(10^k + \lambda)$-dim vector, as shown in Equation. (S26).

$$\mathbf{V_{PseSSC}} = [\mathbf{v_1}, \mathbf{v_2}, \cdots, \mathbf{v_{10^k}}, \mathbf{v_{10^k+1}}, \cdots, \mathbf{v_{10^k+\lambda}}] \tag{S26}$$

Where v_I defined as shown in Equation. (S27).

$$\begin{cases} \dfrac{\mathbf{f_m}}{\sum_{i=1}^{10^k} \mathbf{f_i} + \boldsymbol{\omega} \sum_{j=1}^{\lambda} \boldsymbol{\tau_j}} & \mathbf{1 \le m \le 10^k} \\ \dfrac{\boldsymbol{\omega\tau_{m-10^k}}}{\sum_{i=1}^{10^k} \mathbf{f_i} + \boldsymbol{\omega} \sum_{j=1}^{\lambda} \boldsymbol{\tau_j}} & \mathbf{10^k + 1 \le m \le 10^k + \lambda} \end{cases} \tag{S27}$$

Where $f_m \left(m = 1, 2, 3, \cdots, 10^k\right)$ denotes the normalized frequency of occurrence of structure state combinations of k adjacent nucleobases, ω is the weight coefficient, λ is the maximum number of levels of correlation factors. The first 10^k terms of Equation. (S26) denote the combination information of k-tuple structure states, while the terms from $10^k + 1$ to $10^k + \lambda$ reflect the information of structural order.

The correlation factor τ_j can be defined as Equation. (S28).

$$\boldsymbol{\tau_\lambda} = \frac{\mathbf{1}}{\mathbf{L - \lambda}} \sum_{\mathbf{i=1}}^{\mathbf{L-\lambda}} \boldsymbol{\Theta}(\boldsymbol{\Psi}_i, \boldsymbol{\Psi}_{i+\lambda}) \tag{S28}$$

Here τ_1 is referred to as the first-level correlation factor, representing the correlation between the two nearest contiguous structure state in the nucleotide sequence. τ_2 is referred to as the second-level correlation factor, representing the correlation between two contiguous structure states. τ_3 is referred to as the third-level correlation factor, representing the correlation between three contiguous structure states. τ_4 is referred to as the fourth-level correlation factor, representing the correlation between the fourth contiguous structure states and so forth.

The correlation function $\Theta\left(\Psi_i, \Psi_j\right)$ can be defined as Equation. (S29).

$$\boldsymbol{\Theta}\left(\boldsymbol{\Psi}_i, \boldsymbol{\Psi}_j\right) = \left[\mathbf{F}(\boldsymbol{\Psi}_i) - \mathbf{F}\left(\boldsymbol{\Psi}_j\right)\right]^2 \tag{S29}$$

Where $F(\Psi_i)$ denotes the MFE of the secondary structural state at nucleotide sequence position i, while $F\left(\Psi_j\right)$ denotes the MFE at position j. The values Ψ_i and Ψ_j denote the secondary structural states at positions i and j, respectively.

Reference

46. Dong, Q.; Zhou, S.; Guan, J. A new taxonomy-based protein fold recognition approach based on auto-cross-covariance trans-formation. *Bioinformatics* **2009**, *25*, 2655–2662.
